# Neverland or Tomorrowland? Addressing (In)compatibility among the SDG Pillars in Europe

**DOI:** 10.3390/ijerph182211858

**Published:** 2021-11-12

**Authors:** Joana Costa, Diana Cancela, João Reis

**Affiliations:** 1 DEGEIT—Department of Economics, Management, Industrial Engineering and Tourism, Campus Universitario de Santiago, University of Aveiro, 3810-193 Aveiro, Portugal; link1in@hotmail.com; 2 GOVCOPP—Research Unit on Governance, Competitiveness and Public Policies, Campus Universitário de Santiago, 3810-193 Aveiro, Portugal; p40500@ulusofona.pt; 3 INESCTEC—Institute for Systems and Computer Engineering, Technology and Science, R. Roberto Frias, 4200-465 Porto, Portugal; 4 Industrial Engineering and Management, Faculty of Engineering, Campo Grande, Lusófona University and EIGeS, 1749-024 Lisbon, Portugal

**Keywords:** SDGs, logit panel data, SDG pillars, innovation ecosystems, HDI, entrepreneurial initiative

## Abstract

The 2015–2030 agenda framed Sustainable Development as a Universal venture. This decision has a great historic significance as it encompasses building a better future for the whole of humanity, enrolling the millions who have been denied the chance to live a decent, dignified and fulfilling life and to achieve their potential. For the first time, an entire generation will have the chance to succeed in ending poverty while being the last to have a chance of saving the planet. The world will be a better place in 2030 if humanity succeeds in this journey. However, there is hovering skepticism around the feasibility of this accomplishment. The article aims to empirically test the (in)compatibilities among these objectives even for developed economies such as the European Union countries, demonstrating that unless solid bridges are built promoting innovative networks at a transnational level, welfare and prosperity among those ecosystems will be compromised. The results show that the Sustainable Development Goals (SDG) pillars have heterogeneous determinants, which are to some extent incompatible. Moreover, policy makers need to further reinforce multi-country compensations if the environment is to be preserved.

## 1. Introduction

In recent years, the international community was put at a crossroad of historical proportions. Currently, the world is experiencing extreme challenges, not only concerning climate change, but also many social and economic cataclysms leveraged by the globalized pandemic crisis. Overcoming these problems demands ambitious and concerted efforts by worldwide institutions, governments, policy makers as well as all relevant players providing appropriate responses endeavoring a globalized prosperity and a human centered future.

In 2015, the UN (United Nations) adopted a framework of Sustainable Development Goals (SDGs) to eradicate poverty, promote socioeconomic inclusion encompassing environmental preservation [1]. Notwithstanding, these goals have aroused polarized perceptions, posing on the one hand hope in a better future, and on the other hand severe criticisms for being generalist, broad, inconsistent, unquantifiable and unachievable [2].

Moreover, the recent events in worldwide economies have changed market forces, causing unexpected shifts in both demand and supply. Policy makers and decision makers have the obligation to protect and improve the planet for the sake of all its current inhabitants, while taking the responsibility to deliver a better environment to future generations and not withholding the ability to grow to the poorer [3,4].

All over the world, nations state that they are committed to this effort, coordinating actions and regulations towards the shift for a sustainable world, building a new reality. As a consequence, SDGs became the plan of action for this achievement [4,5,6].

These objectives, endorsed by all UN member states in 2015, represent an ambitious plan for sustainable human development by the year 2030 [5,6]. This universal agenda demands low-, middle- and high-income country involvement. It lays out a set of comprehensive goals that focus on people, the planet, prosperity, peace and partnership for the next 15 years [6,7]. It integrates the vision of “leaving no one behind” which can likely be accomplished through expanded partnerships for both traditional and nontraditional actors such as governments, civil society, the private sector, and the United Nations system [7,8]. Hence, there is a plethora of scientific evidence showing that present tendencies relating to population growth, resource endowments, and economic models cannot stand much longer [9]. The planet faces over occupation and overconsumption; finite resources cannot continue supplying the increasing demand [10,11].

Since sustainable development is a global, multidimensional and multidisciplinary goal, it requires investments being organized in a multi-layer framework [12]. Thus, different governments have associated the different SDGs with specific public policies [13]. Public budgets have been seen as powerful to meet the 2030 Agenda and, therefore, their integration with the goals is approved by the United Nations Development Program (UNDP) [14].

Given the gigantic dimension, complexity of implementation and measurement, of the new agenda, it requires a revitalized Global Partnership to ensure its feasibility [15]. Moreover, given the country heterogeneity, these targets will have to address singularities and constraints present in each economy [16]. This partnership will work in a spirit of global solidarity, in particular solidarity with the poorest and with people in vulnerable situations [8]. It will facilitate an intensive global engagement in support of implementation of all the goals and targets, bringing together governments, the private sector, civil society, the United Nations system and other actors and mobilizing all available resources [7]. The agenda, including the SDGs, can be met within the framework of a revitalized Global Partnership for Sustainable Development, supported by the concrete policies and actions as outlined in the outcome document of the third International Conference on Financing for Development [5,7].

Each country has primary responsibility for its own economic and social development and a secondary responsibility for the enrolment in a globalized network to share with other good practices and knowledge [17,18]. The new agenda deals with the means required for implementation of the goals and targets. The achievement of these targets includes the mobilization of financial resources as well as capacity building and technology transfer of the cutting-edge technologies to environmental preservation to developing and undeveloped countries on favorable terms, including on concessional and preferential terms, as mutually agreed [8]. Public finance, both domestic and international, will play a vital role in providing essential services and public goods, efficiently allocating the financial resources; the entrepreneurial sector, and the civil society organizations will also matter in the implementation of the new agenda [6].

The proposed SDGs are often believed as too ambitious as they concentrate on environmental issues, as well as economic inclusion throughout inequality minimization, while relying upon economic development to foster this civilizational shift promoted in a sustainable ecosystem. The prior eight millennium goals were considered as focused and achievable; hence, these 17 SDGs are believed to be comprehensive and broad [19], becoming eventually unfeasible [20,21].

The international public finance will also enhance these initiatives complementing the efforts of countries in the domestic mobilization of public resources, especially in the poorest and most vulnerable countries with scant endowments of resources. Financial institutions must support policy actions of each country, with special emphasis in developing countries [22]. Governments and public institutions must harmonize their efforts in the implementation with regional and local authorities, sub-regional institutions, international institutions, academia, philanthropic organizations, volunteer groups and others [23].

The exponential increase in the worldwide population, the strive for increased material craves, and increased living standards, and the consequent devastation of natural resources have increased a general conscience for the emergency of sustainability in living styles as well as planning, projecting, developing and maintaining the productive activities [10,11]. Sustainable practices are present in almost all institutional and organizational agendas [24], triggered by greedy demand forces coming from a broader populational sets, supplying greener goods [25] and publicizing sustainable actions [21,26,27].

The sustainability roadmap seems to be an oxymoron as it demands for resource preservation while raising social affluence [1,9,27]. The academic community is often skeptical about the immeasurability of the indicators [3]. The proxies proposed and appraised present wide heterogeneity, impoverishing their functionality as a measurement tool as well as a guidance for the achievement of the goals [15]. Notwithstanding, the introduction of worldwide aspirational goals seems to seed positive vibes; still, their appraisal needs to be crystal clear, to address the effectiveness of the political actions as well the magnitude of its effects [1,3,9,19]. The soundness of the SDGs delivers future generations a better planet; still, the economic achievements seem to be incompatible with the environmental [28], and the first, a necessary condition for the social [29,30]. Despite the complexity of the assignment, measurability has to become a reality due to the importance of the topic [31], and it became a central effort in all the European countries, being of the spotlight of its political decisions [32]. Additionally, the citizens need to be involved in the decision-making processes and develop solid ties of trust with the institutions to enhance the policy actions towards sustainability [6], fighting for transparency and anti-corruption efforts will further enhance the community towards this path which will grant societies a better future [6,17,20].

The article aims to cast light on these connections and providing recommendations to governments, policy makers as well as other relevant players; as a consequence, the purpose is two-fold: identifying the determinants of SDG achievement and quantitatively measure the effect of the different pillars present in the ecosystem in this pathway. An exploration of this topic using quantitative data for the European Countries has not been conducted so far; this would fulfil an important gap in the empirical literature, working as a robustness check to the theoretical and political frameworks. This would mostly assume that if it does not work in developed and cohesive economies, it is doomed to fail in the less developed economies.

The rest of the article is organized as follows: Section 2 addresses sustainability concepts and pillars, as well as its identified determinants in extant literature following a diachronic perspective. Section 3 relates to materials and methods detailing the database construction and its exploratory analysis. Then, Section 4 encompasses the econometric estimations and the model results to identify the determinants of sustainability achievement. Lastly, Section 5 concludes and proposes some policy insights in this vein as well as some limitations and future research paths.

## 2. Theoretical Background on Sustainability and Its Determinants

### 2.1. Sustainable Development

The concept of sustainability has been linked to the topic of human species’ sustainability since the 1970s [33], it is strongly connected to the catching expression of sustainability and sustainable development [34]. Indeed, sustainable development gained momentum in contemporary discourse almost everywhere, either in the side of practitioners, entrepreneurs, policy makers, and others, being on the spot of research with multiple contributions [35,36]. However, despite its pervasiveness and the massive popularity it has garnered over the years, the concept still seems unclear in both empirical and theoretical terms grasping an important set of perspectives [37,38,39].

Quite consensually, the definition of Sustainable Development relies on the one proposed by the Brundtland Commission report in 1987 called “Our Common Future”, underlying on the possibility of delivering the citizens the satisfaction of their present needs without endangering the ability of the next generation do survive and organize a dignified living [7,8]. However, improving living standards while generating prosperity without endangering the environment as well as the ecosystem may sometimes cause disequilibria with the elements as well as the other living creatures in the ecosystem [40].

The conceptualization of sustainable development is three-fold, focusing on the environmental, the economic as well as the social aspects of delivering the forthcoming generations’ vibrant ecosystem which provide them with a space in which to live. It is a matter of balance, resilience and interconnectedness, which will allow human beings to be immersed in the ecosystem in an empathic rather than aggressive way. Once ecosystems are reinforced, these will regenerate, restoring the factor endowments and, thus, minimizing the biological impact of economic actions, which will serve the present and the future necessities [41].

On the one hand, it implies the use of renewable natural resources in a way that does not eliminate or compromise the usefulness of future generations. On the other hand, it implies the use of non-renewable resources in a way that does not imply the access denied by future generations [42].

Over the years, the expansion of humanity has brought a great continuum of unsustainability that has led to the “socio-ecological collapse”, where scarce natural resources collided with the unlimited needs of society [43]. To face the unsustainability felt in society, some efforts were made, namely the creation of international goals, such as SDGs. Their history began in the last century in 1992 through Agenda 21, which aimed to achieve sustainable development in order to improve human life and protect the environment. In 2000, the Millennium Development Goals (MDGs) emerged to eradicate poverty by 2015, leaving socio-economic problems behind. With several changes and renewals, in 2015, the 2030 agenda was adopted, which includes the 17 SDGs to be fulfilled until 2030, which thus replaced the MDGs, contemplating the social, economic and environmental dimensions [6,8]. These goals are composed of 169 targets to be achieved by 2030, measurable with 247 indicators [44].

The European Commission guarantees that the 17 goals were created with the intention that their achievement leaves “no one behind” [6,8,32]. The ecosystem claims that, for moving beyond the rhetoric layers and launching a more substantial agenda for sustainable development, in this vein, a clarification about the conceptual domain, as well as its key pillars, is required [45]. In this vein, the academic debate on the topic grasps multiple dimensions and approaches, and the boundaries of the conceptual definition as well as the related concepts is divergent, e.g., [17,46,47], these controversial perspectives endanger application and measurement of the SDGs. Furthermore, fiercer critics claim the concept is useless if improperly defined; critics also question the indelible tie to wealth generation [47,48].

The generalized debate on the topic grasps, on the one hand, positive feedback due to its societal involvement and a generalized awareness; still, on the other hand, there is much debate on the definitional boundary disregarding the focus in the achievement, which is quite consensual. Additionally, it fails to contextualize the problematic in terms of both time and space, as well as speed of adjustment [49]. In a broad perspective, it seems undeniable that, when sustainable, the system overcomes and withstands difficulties delivering prosperity for the embedded agents [46,50,51]. Notwithstanding, the promotion of welfare is intertwined with higher consumption levels and, therefore, growth. The present economic model demands a never-ending list of assets being placed in the market forcing towards the depletion of resource endowments. Prosperity is a material concept, related to effluence, being material [51,52].

The three-pillar conception of sustainability has become ubiquitous in society [12], encompassing several updates; the concept of sustainable development began to be highly disseminated, being inevitably present in any important political, business or strategic document [42]. This approach presents distinct but interconnected pillars, namely the environment, economy, and society. Decision makers need to visualize the relations, the interconnections and complementarities as well as the trade-offs among them towards the achievement of responsible behavior and the consequent actions in the multi-level sets of communities, in which individuals and communities play a determinant role upholding this target in favor of humankind.

The state of the art evidences the insufficiency of the involvement of the ecosystem players, namely the UN, central and local governments, organizations, and civil society. These agents need to improve the quality of the policies, education, regulations, resource management to promote a conscious action in the journey of sustainability [29].

Necessarily, the assessment of the achievements will be dynamic, varying in time and space, respecting the intergenerational justice. Further clarification is required about the timings and costs as well as the implementation of sustainable actions, and perhaps the acceptance of two complementary branches, “weak and strong sustainability”, relating to economic and non-economic targets [15]. Notwithstanding, SDGs and targets are integrated and indivisible, global in nature and universally applicable, considering different national realities, capacities and levels of development and respecting national policies and priorities. Targets are defined as aspirational and global, with each government setting its own national targets guided by the global level of ambition in national circumstances. Each government will also decide how these aspirational and global targets should be incorporated into their nation.

### 2.2. SDG Pillars

Supranational institutions have proposed and agreed on the establishment of SDGs to reverse the detrimental consequences of the Anthropocene era—a scene in which human domination performs the dismemberment and degradation of the biosphere—and eradicate societal problems [1,10]. Notwithstanding, humanity is facing a jigsaw of extreme challenges, such as extreme poverty, poor health and education, social and political inequality, ineffective policies and governance, unsustainable population growth and resource use, changing climate, and declining biodiversity [4,9].

For a long time, the vision about sustainability was very holistic and vast. However, the three complementary pillars, also known as triple bottom line, were created by Elkington [53,54], which account for three important dimensions of sustainability: profit (economic sustainability); planet (environmental sustainability); people (social sustainability) [10,12]. The distribution of sustainability was also driven by the growing concern of society and companies for the environment and the social level felt in the world, with a focus on future generations [55].

Sustainability is, therefore, commonly agreed to be a three-pillar framework; however, it can also be appraised as a bidirectional connection between humans and the ecosystem. Despite the enormous progress achieved by technological innovation and its new products, ensuring a minimum quality of the elements such as water, air or land is a responsible action for the present productive structures and also the future [10]. In this vein, it is important to consider the pertinence of these vectors [53,54], and also their interdependence. Bringing the people social sustainability seems to be unfeasible without economic sustainability. Additionally, respect for environmental sustainability will grant economic sustainability for other parts in the ecosystem. These connections must address the underlying purposes of entrepreneurial endeavors—the creation of sustainable value [56,57].

The classical purpose of business organizations is strongly tied to purely economic imperatives, relating to the efficient use of the factors of production [58,59]. The emergence of environmental sustainability forces firms to consider an additional dimension of organizational performance, even though this entrepreneurial focus is being increasingly scrutinized by other agents in the ecosystem [9,56,57]. Profit maximization per se does not encompass environmental objectives such as efficient energy consumption, reduction in carbon footprint, or provision of ecologically sustainable solutions. Indeed, the classical managerial strategy was concentrated in the devil’s quadrangle: time, cost, quality, and flexibility [56,58,59]. Hence, over recent decades, sustainability has become an important emergent role in the management of business processes [12,57,58,60].

#### 2.2.1. Economic Sustainability

After World War II, society felt an urgent need to join international efforts to help the development of less advanced countries. It was at this time that the concept of economic development began to emerge, reflecting the increase in the material well-being of society, which led to an increase in income per capita, thus being directly related to economic growth [12].

Obtaining sustained, inclusive and sustainable economic growth is essential for prosperity. This will only be possible if wealth is shared and income inequality is addressed. Working to build dynamic, sustainable, innovative and people-centered economies, promoting youth employment and women’s economic empowerment, in particular, and decent work for all [8]. Cohesive nationally owned sustainable development strategies, supported by integrated national financing frameworks, will be at the heart of our efforts. We reiterate that each country has primary responsibility for its own economic and social development and that the role of national policies and development strategies cannot be overemphasized [16].

Later, the emergence of environmental movements, as well as the occurrence of natural disasters, called into question the economic growth of several countries, intensifying the need for “economic development” [49]. More recent aspects, such as the economic and financial crisis, helped to define more rigorously the economic pillar of sustainability, emphasizing “economic progress”. In this vein, the economic evaluation disregards the environmental facet [42]. In the last five decades, the pillar of economic development has received the most attention, with the reconciliation of the sustainability and economic growth of countries being a challenge [7,42].

Private business activity, investment and innovation are major drivers of productivity, inclusive economic growth and job creation. We acknowledge the diversity of the private sector, ranging from microenterprises to cooperatives to multinationals. We call upon all businesses to apply their creativity and innovation to solving sustainable development challenges. We will foster a dynamic and well-functioning business sector, while protecting labor rights and environmental and health standards. Overall, economic sustainability “implies a production system that meets current consumption levels without compromising future needs” [29].

#### 2.2.2. Social Sustainability

In the existing literature, it has been verified that the definition of social development is not so clear. Very often, it is considered as “a positive condition within communities, and a process within communities that can achieve that condition” [54]. However, it is said to be “the extent to which social values, social identities, social relationships and social institutions can continue into the future” [42]. Still, Torjman [33] states that “From a social perspective in particular, human well-being cannot be sustained without a healthy environment and is equally unlikely in the absence of a vibrant economy”, linking the social to the economic and environmental dimensions. Considering the various definitions of social sustainability existing in the literature (although with several divergences among authors), it can be said that this pillar is associated with the networks created among people in society through their participation in social groups and activities [60,61]. There is a consensus that in order to achieve full social sustainability, society must comply with the principles of equality, promoting an equal distribution of opportunities among people [61].

However, social sustainability is a highly important dimension which has been forgotten in comparison to other sustainability pillars [30]. Some indicators that reflect social sustainability are justice, security, education, and health [13,54]. Since these are very intangible indicators, this pillar becomes difficult to measure and predict [47].

To the branch of more skeptical academics, the SDGs included in this category are, to some extent, unrealistic, because they ignore the realities of human behavior, or they consider the human being with a positive and altruistic essence, which is not the case. Aiming to “achieve gender equality, social inclusion, and human rights for all” and “transform governance for sustainable development” are fundamental values and noble ideals; however, they have not been achieved in all of the human history [9].

#### 2.2.3. Environmental Sustainability

According to Moldan et al. [42], “Sustainable development used to be more or less understood as social and economic development that should be environmentally sustainable”. Thus, although the focus is most often on the economic and social dimension, the third pillar of sustainability linked to the environment must be truly understood. For Moldan et al., environmental sustainability is “maintaining nature’s services at a suitable level”. The concept of environmental sustainability calls on society to live and consume natural resources (such as land, water, fuel, etc.) at a sustainable rate, without compromising the well-being of future populations with a scarcity of natural resources [53]. Often, the environmental pillar is seen as the main one of the three because if the carrying capacity of the environment is low, it will compromise the social and economic system [13,54].

To Mensah [29], “The effects of climate change, for instance, provide a convincing argument for the need for environmental sustainability”. Thus, climate change whose effects are felt in society in the form of warming of the oceans and atmosphere, sea level rise, increase in greenhouse gas emissions, etc., lead society to think about the pillar of environmental sustainability with urgency.

In sum, the correspondence between pillars and SDGs is described below (Table 1):

### 2.3. Harnessing Innovation to Leave No One Behind

Innovation has been a buzzword. It has been a response to various current problems such as globalization, political transformation, technological revolutions, environmental changes, etc., thus emerging within all sectors in the form of products, services and processes. Innovation has been core in programs, supported by governments and the private sector, often integrated into sustainable economic development plans [62].

Technology and innovation to achieve SDGs have been fostered, promoting coordination, coherence and cooperation within the UN system on science, technology and innovation, enhancing synergies and efficiency, in particular to enhance multi-agent initiatives, is bound with ecosystems [8,17]. However, reporting on sustainability is still largely voluntary, apart from aspects that have already been regulated by law such as the emissions trading acts. The community should understand more about entrepreneurial practices and their responsible action to promote the welfare of the community while maximizing profit [63]. When setting sustainability objectives, it is essential to design an action plan focusing on quantitative measurements with concrete targets, which encompasses all dimensions of sustainability. The journey to sustainable development in the corporative field can only be paved when it becomes legally compulsory to contribute to sustainability in a tridimensional way.

**Hypothesis** **1** **(H1).** *Higher innovation levels will raise the probability of achieving the SDGs*.

### 2.4. Entrepreneurial Ecosystem Synergies

If humanity does not build a sustainable productive environment to provide for society, and if the endowments of resources are not about to be preserved, it will become impossible to maintain the social organization as we know it. A sustainable ecosystem also depends on a sustainable society and a sustainable productive organization. With this mindset, economic players are interconnected, such that firms need to conceive of new ways to co-operate with their suppliers, customers, and other stakeholders—including competitors—while ensuring that their benefits are not limited to corporate citizenship and generating competitive advantages [25,53,59].

Entrepreneurship is cited as a vehicle for this transformation from unsustainable to sustainable [58]. In recent years, there has been a growing discussion in the existing literature about the connection between the entrepreneurial ecosystem and sustainability. In addition, the importance of entrepreneurship as a path to sustainability has recently been addressed in both academic research and government and business practices [63].

There are many authors who propose entrepreneurship as a solution to environmental, social and economic issues; the study by Dhahri and Omri [58] shows that the economic and social dimensions of sustainable development are positively associated with entrepreneurship, while the environmental dimension is negatively affected by it. In general, the existing literature points to a positive connection between these two major areas of action. However, although entrepreneurial activity is seen as a key tool for achieving sustainability, there is still a high degree of uncertainty, thus making it interesting to study this relationship [58,59].

**Hypothesis** **2** **(H2).** *Entrepreneurial vibrance will raise the probability of achieving the SDGs*.

### 2.5. The Role of Prosperity in Sustainability

The Human Development Index (HDI) corresponds to an indicator created in 1990 that changed the perception of development as well as the way in which it is measured [64]. This indicator shows itself as a multidimensional alternative to Gross Domestic Product (GDP) [65]. While GDP only allows economic development to be measured and analyzed, the HDI is composed of dimensions of income, health and education. Although it is a very broad indicator, the existing literature shows that it does not consider the environmental dimension and, therefore, cannot fully explain sustainable development (which incorporates the economic, social and environmental pillars) [62]. However, the appearance of this indicator spurred the creation of the Millennium Development Goals in 2000 and, later, the most updated version, Sustainable Development Goals [64,65].

Over time, some efforts have been made to update the HDI so that it can encompass the three pillars of sustainability. Thus, indicators have been created that address weak sustainability—characterizing the three pillars as replaceable—and strong sustainability—where the three dimensions are complementary [66].

According to Sagar and Najam [65], “the HDI can be considered as a first and important step toward incorporating broad notions of sustainability into measures of development”. Therefore, although the HDI does not consider the environmental dimension, it is positively correlated with sustainable development [67].

Consumerism and its rules lead to the oversupply and overconsumption of many consumer products; on the other hand, companies aiming to fulfill their greedy profit maximization strategies compete to identify services and product features that will attract customers, accelerating obsolescence and creating vain utility. To serve this purpose, the world has passively assisted to the rise of business analytics and customer service/assistance management to establish continuous flows of information. Shifting to a new economic paradigm, embedded in sustainability, forces the players to assume that the dominant issue is mitigating environmental impacts, promoting responsible innovations so that, to some extent, that it becomes bearable to inhabit planet earth. In this vein, one of the most significant environmental trends in recent years has been the “greening” of the marketplace, pulled by the emergence of the “green consumer” [19,26].

**Hypothesis** **3** **(H3).** *Higher HDI levels will increase the chance of achieving the SDGs*.

## 3. Materials and Methods

### 3.1. Database

To perform the empirical part of the investigation, original data from Eurostat, Transparency International, Country Economy and European Innovation Scoreboard were combined for different countries between 2011 and 2017. The collection of secondary data from supranational institutions reinforced the reliability of the information gathered, as well as the replicability and enlargement by future research.

Eurostat is the statistical office of the European Union partnering with national statistical institutes and other national authorities across member states (the European Statistical System), as well as other countries belonging to the European Economic Area and Switzerland (OECD/Eurostat, n.d.). Additionally, Transparency International data [68] allowed some missing information in Eurostat to be completed regarding the Corruption Perception Index, namely for the year 2011 (results were homogenized with those from the Eurostat). Additionally, the HDI was collected from the Country Economy database for each country in the sample. Lastly, EIS was used to collect data for the entrepreneurial ecosystem, aiming to provide a comparative analysis of innovation performance in EU countries, other European countries and regional neighbors.

The methodological option was to build a balanced panel for carrying the econometric estimations with the avoidance of biases. This forced the removal of countries with excessive missing data, as well as variables with scant information. As a consequence, due to the inexistence of data, Albania, Bosnia, South Korea, United States of America, Japan, Kosovo, Lichtenstein, Montenegro, Russia, United Kingdom, Northern Macedonia, Malta and Switzerland were removed from the complete Eurostat list, leading the analysis to 29 territories (listed in Table 2). This removal allowed keeping an important and representative variety of geographies in the EU as well as the representative SDGs in each pillar (details in Table 1) and their expected determinants. The fully balanced panel, therefore, encompassed data from 2011 to 2017, totaling 203 observations.

Due to severe availability constraints in data (almost all variables in the goal missing), a selection of the SDGs was made, removing SDG 6 (clean water and sanitation), 14 (life below water), 15 (life on land) and 17 (partnerships for attaining the goals). This decision was made as the low availability of data could jeopardize the robustness of the econometric analysis to be carried out; additionally, all pillars were still represented (social sustainability with 7 goals, economic sustainability with 5 and environmental sustainability with 1—climate action which is the more homogeneously measurable). Punctual missing values in the series were fulfilled with the conventional moving average method. In other words, orthodox statistical methods were used to keep the broader panel which granted robustness and validity to the estimation.

### 3.2. Variable Description

Given that the database in use encompasses multiple data sources as well as new variables based on the original, Table 3 details their characteristics in the clearest and most understandable way. The first four variables aim to proxy our endogenous variable, sustainability, per pillar and in general; the following are predictors aiming to address the role of innovation in the promotion of sustainability (and test Hypothesis 1), entrepreneurial vibrance (Hypothesis 2) and, lastly, the importance of the stage of development (Hypothesis 3).

As the purpose of the study was to identify enhancers and hinderers to the achievement of sustainability in its multiple dimensions, the methodological option chosen was to transform the continuous variables into binary. As a consequence, country achievements for each dimension were ranked, given the value 1 if the result overcomes the average threshold or 0 if it did not.

Furthermore, to carry out the binary variables related to sustainability both in general terms and in each of the three pillars, all indicators in the goal were encompassed and ranked to determine the final country positioning. Then, the ranking of each country for each variable that explains each pillar of sustainability was carried forward, to build the overall performance. Given that most of the variables relating to the SDGs need to be appraised in comparative terms rather than as an absolute figure, a comparison to the overall average was performed. Thus, if the average for each country was lower than the global average, the country was given a score of 0 (did not reach the SDG); if the average for each country was higher than the global average, the country was given a score of 1 (reached the SDG). Then, carrying forward the binary variables produced for each SDG, and given that each pillar was considered as a count of items included in each pillar, the country will be given score 1 when reaching at least half of SDGs inserted in each pillar. This procedure allowed any bias caused by a different number of goals included in each pillar to be removed. These three binary variables correspond to a dependent variable in each pillar model that will be tested along next sections; an overall variable was built following the same procedure for general sustainability which disregarded the pillars and combined the entire set of goals. Choosing the raw indicators to create a general ranking as the methodological option allowed us to avoid some potential multicollinearity among the pillars.

### 3.3. Exploratory Analysis

Encompassing the predictors described in Table 3, the analysis will be conducted to address the identification and quantification of the contribution of innovation, entrepreneurial vibrance and HDI to the achievement of each of the three pillars of sustainability and global sustainability (accordingly to the stated hypotheses). Thus, four panel data models will be run, firstly for each of the three pillars presented and then to global sustainability:

**Model 1:** The impact of the entrepreneurial and innovative ecosystem and the contribution of different agents in achieving social sustainability;
**Model 2:** The impact of the entrepreneurial and innovative ecosystem and the contribution of different agents in achieving economic sustainability;
**Model 3:** The impact of the entrepreneurial and innovative ecosystem and the contribution of different agents in achieving environmental sustainability.
**Model 4:** The impact of the entrepreneurial and innovative ecosystem and the contribution of different agents in achieving global sustainability.

Given that the sample was included in several time frames, it became possible to combine the country dimension (cross section) with a diachronic perspective (time series), consequently performing panel data analysis. However, rather than presenting the correlations for the entire time span, including the entire dataset, only the most recent year was chosen. This methodological choice was made to evidence the sectional correlation strength rather than the time inflated value. Thus, as the four models will be run with the same predictors, descriptive statistics and correlations were presented covering only one of the pillars, in this case for social sustainability (SOC_SUST), and the year 2017 (no significant differences were found when running the same statistics for the other dependent variables) (Table 4).

Regarding correlation, moderate and high intensities are found, being able to provide evidence for the existence of a multicollinearity between predictors. Therefore, in the next section, the values of Variance Inflation Factor (VIF) are presented to conclude an as-yet inexistant classic hypothesis violation. These results were considered for the model’s construction in the following section.

## 4. Econometric Analysis

### 4.1. Econometric Estimation

To carry out econometric analysis, in addition to the construction of databases in Excel, SPSS and Stata were used in order to provide the necessary outputs. Thus, this section aims to describe the results obtained. Additionally, a detailed data analysis will be provided in the following section.

As mentioned above, given the strong correlations between the predictors that will be used in the four models to be analyzed, it is important to carry out a robust analysis that allows the (in)existence of multicollinearity in the models to be verified. It should be noted that multicollinearity occurs when two or more predictor variables of the models are strongly correlated with each other, leading to their failure to provide independent information. To confirm its (in)existence, the Pearson correlation coefficients can be analyzed; however, the VIF test allowed robust conclusions to be achieved on the inexistence of multicollinearity among all variables with the exception of corruption. As a consequence it was removed from the estimations to guarantee the non-violation of the classical hypotheses and, consequently, estimation validity. Thus, three linear regressions were run for 2011 and 2017, encompassing the binary variables associated with each pillar of sustainability, with the sole purpose of verifying the results of the VIF test. Thus, considering the conventional thresholds, VIF values greater than 8 indicate that the predictor has multicollinearity in the model and, therefore, suggest it does not have sufficient statistical robustness. The following results were obtained using SPSS software (Table 5):

In the previous table (Table 5), we can see that the variable concerning the corruption perception index (CORR_PIND) presents a VIF of 8.804 for the three pillars in 2011 and, therefore, may compromise the robustness of the models. Thus, this variable is excluded from the initial set presented in the codebook given the potentially harmful effect in the extant model.

Given the binary nature of the dependent variables (having achieved or not achieved sustainability), a panel model was run, using in the logistic form. As the coefficients in logistic regressions are not directly interpretable, the results of panel data in logistic form were omitted. Table 6 presents the marginal effects that quantify the impact on achieving sustainability in the three pillars and the overall target caused by unitary changes in the independent variables. These coefficients show the change in percentage points operated in the endogenous variable caused by a caeteris paribus unitary change in the predictor [69].

In the four models, the predictors are purposefully kept constant to allow for direct comparisons between pillars and the magnitude of impacts. Thus, it is possible to understand that the different determinants of the achievements do vary in terms of the magnitude and the direction of the effect, leading to a preliminary insight that these targets may become unfeasible in time.

### 4.2. Econometric Results

Model 1 shows the determinants of social sustainability. Quite surprisingly, the level of education fails to be statistically significant in the propensity to achieve this sustainability pillar, as well as the innovation related determinants (PA_EPO and RD_GDP). Another interesting finding is the negative effect of the average life expectancy, potentially evidencing the drawbacks of population ageing in social sustainability. Reinforcing the previous expectations, entrepreneurial vibrancy has a very positive impact in social sustainability, demonstrating the importance in fomenting the entrepreneurial initiative to achieve socially related targets. In the same vein, improving the living standards through the HDI is also a determinant in the accomplishment of these goals. In sum, the improvements in transparency, justice and other social dimensions of sustainability will be strongly connected to the different forms of work and the entrepreneurial ecosystem as well as the welfare achieved throughout the improvements in the HDI.

In regard to the economic sustainability drivers, important differences are found as the only significant predictor is the HDI; this means that all the designed goals in this pillar will be leveraged by the improvements in income generation, education levels and sanitation. This result is quite unsurprising as the eradication of essential needs can easily be achieved in societies able to generate enough income to be distributed across the population. However, the insignificance of other coefficients can be interpreted as a warning; potentially, the targets were designed in a short-sighted way, or do not demand for harmonization with other dimensions of social dignity and respect for the environment. It seems that these objectives are merely income related and the aims of this agenda are far more ambitious.

The most surprising findings are in Model 3; however, their interpretation needs to be careful, and perhaps demands a deeper analysis of the results. Innovation seems to be to be determinant in this vector as the two predictors relating to the innovation ecosystem are significant. When appraising the impact of innovation outputs, throughout the patent predictor, the model shows a that each additional patent raises the chance of achieving environmental sustainability by 2.2 percentage points. However, the RandD-to-GDP ratio has a negative impact on Environmental sustainability. At first glance, this effect could make no sense; still, it is important to consider that the vast majority of research efforts by firms is connected no non-product innovations (they relate to other innovation types which will lead to cost minimization), and some research efforts are not ecologically driven. Therefore, it seems relevant that policy makers should be aware of the regulations to be implemented to re-direct the research efforts towards environmentally friendly targets.

Another aspect to be taken into consideration is the negative impact of raising higher education levels. This result may demonstrate that highly skilled workers tend to find occupation in industries that endanger the environment. Further analysis should be made for this point to fully understand the side effects of this inverse relation.

When appraising sustainability as a whole—encompassing the three pillars together (Model 4)—the most important predictor is the HDI. This result further reinforces the previous discussion and results, evidencing some bias towards an economic perspective and disregarding social and environmental vectors.

These results provide evidence that the pillars would be achieved better if approached sequentially rather than considering the agenda as an “all-in” goal.

## 5. Conclusions

It is undeniable that sustainability is one of the most demanding challenges of our time and can no longer be postponed. Society must urgently address the Malthusian–Darwinian cycle, given the natural bias of all organisms in favoring themselves and their groups over others [24,49].

All over the world, countries state being committed to sustainability in all its dimensions. The UN project is a collective effort to save humanity and the planet. People, planet, prosperity, peace, and partnership are the pillars of the agenda [5,6,8].

As a final remark, and based on the empirical results, one can state that there is an urgent need of a careful, rigorous and effective science-based analysis of the strategical tools implemented. Given the growing climate and environmental threats, there are indeed ways to harmonize material effluence with environmental preservation [18]. It is worth considering that addressing some SDGs may endanger others [9]. As a consequence, sustainable development is at risk of becoming a cliché, to whom everybody mentions but nobody seems to define, improve or implement [29,45].

Transnational institutions have recognized that the target is challenging; still, there seems to be no plan B to make sure that there is a future for the generations to come. Still, mid-income countries will face significant challenges to meet these objectives, which were made harder with the pandemic crisis due to the struggles in most macroeconomic scenarios. The ongoing objectives demand structural changes, mostly in what concerns income generation and distribution. The developed world seems to play a central role in this process as there is a need to accept knowledge sharing not as an altruistic act, but an egoistic act. Less-developed countries must be pushed to responsible innovation paths if humanity wants to keep the planet as a place we can live in.

The present generation has a duty to build a world with widespread economic progress, plenty of social trust, with inclusion and diversity apart from the scourges caused by human action. It is undeniable that the future must be human centered. At present, the pillars are far closer to the conventional paradigms on classical income generation rather than resource preservation. According to the extant literature, the vast majority of the goals are placed under the social sustainability umbrella; however, it seems difficult to eradicate poverty, hunger and improve health without income generation. If the world really pursues these points, the countries lagging behind need to be helped, as their prosperity path needs to be fast as well as environmentally friendly. Moreover, without the removal of these basic problems, the other objectives are endangered. It seems that they are some sort of a ladder. Starvation and illiteracy seem to prevent justice and equality.

Moreover, economic sustainability needs to be strongly rooted in innovation practices. Once more, developed nations need to consider knowledge sharing as a positive externality to their individual efforts, as the improvements in energy and production will benefit all. Notwithstanding, there is a need to develop policy action aiming to compensate these knowledge frameworks and the promotion of a solid globalized ecosystem.

Given the unexpected result in terms of life expectancy and social sustainability, the problem can be uploaded to the population ageing problem, and here, poorer countries have the advantage.

Hence, the empirical results evidence that the measures aiming towards stopping climate change seem to be disconnected to the economic sustainability. This is an important flaw in the overall strategy needing for further analysis. If we accept that the only way to avoid climate change is by means of thwarting the use of the endowments of resources, we are condemning to poverty half of the planet.

The globalized achievement of a dignified, prosperous and fulfilling existence to all human beings requires a responsible exploitation of resources. The developed world needs to understand the importance of its heritage and share its expertise in resource preservation for the sake of the entire planet. If these flows do not exist, poorer economies cannot afford to preserve their resources, as they face poverty. On the other hand, responsible innovation practices must be adopted in the developed economies as the future depends on them.

The present research encompasses a limited set of European countries. Therefore, there is a need for a future enlargement of the analysis to consolidate the present findings. The time span is relatively short; however, it uses the full set of data available in the present. Future research may address in detail each of the pillars and could consider other proxies to understand in detail the underlying enablers and hinderers of these goals.

There is a growing debate on the importance of these pillars and the urgency for constructing a human-centered paradigm. The proposal of a sustainable personality adds on top of the extant vector the ethical and civic dimension, moving from formal education to values and actions [70]. Perhaps some hindering factors to the achievement of the SDGs strongly ties into the full achievement of frugal habits and responsible actions assuming the shared values with natural balance.

Another aspect demanding additional attention is the connection of these objectives with the current populational trends as the targets can be misleading [71]. Additionally, migratory flows can no longer be neglected. Future equilibria need to encompass these constraints as demography may be key to achieving different positioning.

However, the empirical results achieved by the models in analysis seem to point towards a pyramidal organization of the goals, rather than a simultaneous achievement, and the top of the framework will be reached by the less favored if the wealthier nations do understand that there is only one environment, and the delay of prosperity in some parts of the world will give those countries no alternative than to fully exploit all the natural resources, disregarding the responsible innovation.

Our aim was to understand the extent to which the UN’s plan was feasible, given its centrality for the near future of the entire world. Given the emergency of its implementation, policy makers should consider some fine-tuning adjustments as well as some prioritizations in the goals, as humanity cannot afford to fail this agenda. The COVID-19 pandemic did put some of these targets on hold, and reorganized the diplomatic arrangements in regard to the decisions to be made. Still, the survival of the human species seems to take priority over the political dimension.

## Figures and Tables

**Table 1:** SDGs in use per pillar.

Social Sustainability	Economic Sustainability	Environmental Sustainability
(1) No Poverty	(7) Affordable and Clean Energy	(13) Climate Action
(2) Zero Hunger	(8) Decent Work and Economic Growth	
(3) Good Health and Well-Being	(9) Industry, Innovation and Infrastructure	
(4) Quality Education	(11) Sustainable Cities and Communities	
(5) Gender Equality	(12) Responsible Consumption and Production	
(10) Reduced Inequalities		
(16) Peace, Justice and Strong Institutions		

**Table 2:** Countries included in the database.

	Countries Analyzed	
Austria	Croatia	Romania
Belgium	Hungary	Sweden
Bulgaria	Ireland	Slovenia
Cyprus	Iceland	Slovakia
Czech Republic	Italy	Turkey
Germany	Lithuania	Finland
Denmark	Luxembourg	France
Estonia	Latvia	Poland
Greece	Netherlands	Portugal
Spain	Norway	

**Table 3:** Variable description, sources and measurements.

Variables	Description	Source	Link	Measurement
SOC_SUST	Social sustainability	Eurostat		Binary
ECO_SUST	Economic sustainability	Eurostat	https://ec.europa.eu/eurostat/data/database (accessed on 3 October 2021)	Binary
ENV_SUST	Environmental sustainability	Eurostat		Binary
SUSTAINABILITY	Sustainability	Eurostat		Binary
PA_EPO	Number of patents filed with the European Patent Office	Eurostat	https://ec.europa.eu/eurostat/databrowser/view/sdg_09_40/default/table?lang=en (accessed on 3 October 2021)	Count
CORR_PIND	Corruption Perception Index	Eurostat + Transparency International	https://ec.europa.eu/eurostat/databrowser/view/sdg_16_50/default/table?lang=en (accessed on 3 October 2021) https://www.transparency.org/en/cpi/2020/index/nzl (accessed on 3 October 2021)	Index
HED_L	High education level	Eurostat	https://ec.europa.eu/eurostat/databrowser/view/sdg_04_20/default/table?lang=en (accessed on 3 October 2021)	Percentage
RD_GDP	Gross internal expense on RandD	Eurostat	https://ec.europa.eu/eurostat/databrowser/view/t2020_20/default/table?lang=en (accessed on 3 October 2021)	Percentage
ALE	Average life expectancy	Eurostat	(https://ec.europa.eu/eurostat/databrowser/view/tps00205/default/table?lang=en) (accessed on 3 October 2021)	Discrete
HDI	Human Development Index	Country Economy	(https://countryeconomy.com/) (accessed on 3 October 2021)	Index
ENTREP	Opportunity-Driven Entrepreneurship	European Innovation Scoreboard	(https://data.europa.eu/data/datasets/european-innovation-scoreboard-2019?locale=es) (accessed on 3 October 2021)	Index

**Table 4:** Descriptive statistics and correlations.

VARIABLES	Mean	S.D.	Min.	Max.	(a)	(b)	(c)	(d)	(e)	(f)	(g)	(h)	(i)	(j)
**SOC_SUST (a)**	0.55	0.51	0	1	1									
**PA_EPO (b)**	2252.62	5080.81	10	25,539	0.288	1								
**CORR_PIND (c)**	62.79	15.44	32	88	0.568 **	0.369 *	1							
**HED_L (d)**	42.29	9.42	26.3	58	0.430 *	−0.120	0.501 **	1						
**RD_GDP (e)**	1.62	0.85	0.5	3.36	0.695 **	0.497 **	0.667 **	0.224	1					
**ALE (f)**	80.09	2.77	74.8	83.4	0.314	0.237	0.332	0.142	0.566 **	1				
**HDI (g)**	0.89	0.04	0.811	0.95	0.640 **	0.352	0.679 **	0.615 **	0.766 **	0.562 **	1			
**ENTREP (h)**	3.61	2.63	1.01	11.09	0.543 **	0.158	0.605 **	0.466 *	0.640 **	0.397 *	0.662 **	1		
**ECO_SUST (i)**	0.52	0.509	0	1	0.656 **	0.180	0.578 **	0.370 *	0.706 **	0.295	0.737 **	0.550 **	1	
**ENV_SUST (j)**	0.52	0.509	0	1	−0.316	0.349	−0.036	−0.395 *	0.135	0.038	−0.186	−0.026	−0.243	1

Standard errors in parentheses: *** *p* < 0.01, ** *p* < 0.05, * *p* < 0.1.

**Table 5:** Variance inflation factor.

		2011			2017	
VARIABLES	SOC_SUST	ECO_SUST	ENV_SUST	SOC_SUST	ECO_SUST	ENV_SUST
PA_EPO	1.548	1.548	1.548	1.667	1.667	1.667
CORR_PIND	8.804	8.804	8.804	2.578	2.578	2.578
HED_L	3.000	3.000	3.000	3.031	3.031	3.031
RD_GDP	3.786	3.786	3.786	4.485	4.485	4.485
ALE	1.786	1.786	1.786	1.686	1.686	1.686
HDI	4.722	4.722	4.722	5.784	5.784	5.784
ENTREP	2.794	2.794	2.794	2.208	2.208	2.208

**Table 6:** Econometric estimations—marginal effects after panel data estimation.

VARIABLES	SOC_SUST	ECO_SUST	ENV_SUST	GLOB_SUST
PA_EPO	−0.000 (0.439)	−0.000 (0.670)	0.022 *** (0.000)	0.000 (0.466)
HED_L	0.056 (0.422)	−0.003 (0.977)	−0.444 ** (0.021)	0.052 (0.588)
RD_GDP	1.729 (0.101)	1.419 (0.261)	−11.744 *** (0.009)	2.172 * (0.078)
ALE	−0.385 * (0.100)	−0.356 (0.341)	0.112 (0.918)	0.024 (0.943)
HDI	50.202 * (0.092)	152.488 ** (0.022)	17.434 (0.796)	76.692 ** (0.022)
ENTREP	1.221 ** (0.046)	−0.143 (0.659)	−1.142 (0.186)	0.088 (0.762)

Standard errors in parentheses: *** *p* < 0.01, ** *p* < 0.05, * *p* < 0.1.
